# Comparison of Myeloid Cells in Circulation and in the Tumor Microenvironment of Patients with Colorectal and Breast Cancers

**DOI:** 10.1155/2017/7989020

**Published:** 2017-11-05

**Authors:** Salman M. Toor, Eyad Elkord

**Affiliations:** ^1^Cancer Research Center, Qatar Biomedical Research Institute, College of Science and Engineering, Hamad Bin Khalifa University, Qatar Foundation, Doha, Qatar; ^2^College of Medicine and Health Sciences, United Arab Emirates University, Al Ain, UAE; ^3^Institute of Cancer Sciences, University of Manchester, Manchester, UK

## Abstract

We have previously reported levels of myeloid cells in the periphery and in the tumor microenvironment (TME) of patients with primary breast cancer (PBC) and colorectal cancer (CRC). We found that both PBC and CRC patients have significantly higher levels of granulocytic and immature myeloid cells in the TME. Additionally, we reported an expansion of circulating granulocytic myeloid cells in CRC patients, but not in PBC patients. In this report, we compared levels of myeloid cells between these two common cancers and have added data from more cancer patients. We also investigated associations between clinical stage/histological grade of tumors and levels of myeloid cells in cancer patients. We found that although granulocytic myeloid cells were expanded in the TME of both PBC and CRC patients, the levels of these cells were significantly higher in the TME of CRC patients. Moreover, our results indicate that increased levels of circulating granulocytic myeloid cells are associated with poorly differentiated tumors in CRC patients. Taken together, this work suggests that CRC patients may benefit more from the development of therapeutic agents to promote myeloid cell differentiation or inhibition for the reversal of immune suppression.

## 1. Introduction

Immunosuppressive cells are known to impair antitumor immune responses primarily by inhibiting host T cell responses against tumor antigens. Myeloid-derived suppressor cells (MDSC) and regulatory T cells (Treg) are suppressive cells, which can dampen immune responses and are found elevated in periphery and the tumor microenvironment (TME) of various human cancers to ultimately facilitate tumor progression. Numerous studies have aimed to investigate the levels of these cells in cancer patients in order to target these cells to provoke antitumor immunity and inhibit tumor progression.

Colorectal and breast cancers are two of the most common human cancers worldwide resulting in a combined total of up to 1.4 million deaths globally [1]. Myelopoiesis is a tightly regulated process under normal health, but pathological conditions including cancers can result in the disruption of differentiation of various cellular populations, resulting in generation of highly suppressive cells, halted at varying stages of maturation called MDSC [2, 3]. However, the lack of uniform markers due to their heterogenic nature has made it challenging to identify these cells. Our previous work focused on investigating the levels of cells of myeloid lineage in the periphery and the TME of colorectal cancer (CRC) [4] and primary breast cancer (PBC) patients [5] and comparing their levels in peripheral blood from healthy donors (HD) as controls.

Recent decades have seen great advances in developing novel therapeutic approaches of cancer immunotherapy. Better understanding of the immune profile of the TME and periphery of cancer patients can help in identifying key components of host immune response that may be targeted to revert immunosuppression. We have also recently shown an expansion of highly suppressive infiltrating Treg in the TME of CRC and PBC patients [6, 7]. Therefore, these results could broaden our knowledge on the role of infiltrating and immunosuppressive lymphoid and myeloid populations in the TME of patients with colorectal and breast cancers.

We found that PBC patients have significantly higher levels of myeloid cells with granulocytic morphology (granulocytic myeloid cells; GMC) and immature myeloid cells (IMC) in the TME. Interestingly, this expansion was not reflected in peripheral blood of PBC patients, as the levels of circulating myeloid cells were similar to HD. In contrast, we reported an expansion of GMC in both peripheral blood and the TME of CRC patients. IMC were also expanded in the TME of CRC patients but not in peripheral blood. In this study, we compared levels of myeloid cells in circulation and the TME of patients with two of the most common cancers but presenting with distinct clinical and pathological features.

## 2. Materials and Methods

### 2.1. Patients and Healthy Donor Samples

Peripheral blood samples were collected from 21 HD, 30 PBC patients, and 20 CRC patients who did not receive any treatment prior to surgery at Tawam Hospital, Al Ain, UAE, and Al Noor Hospital, Abu Dhabi, UAE. Tumor and paired, adjacent normal tissue samples were also collected from cancer patients (PBC *n* = 10 and CRC *n* = 11). Written consent forms were signed by all patients and donors prior to sample collection, under ethics approved by Al Ain ethics committee UAEU, UAE (13/23-CRD 244/13).

Patients were divided and compared based on the TNM staging and tumor histological grade. PBC patients with stage I (*n* = 15) were compared with patients with stage II and III cancers (*n* = 15), while CRC patients with stage I and II (*n* = 9) were compared with CRC patients with stage III and IV (*n* = 11). Additionally, patients with tumor histological grade I and II (PBC *n* = 15 and CRC *n* = 16) representing well-to-moderately defined tumor cells were compared with patients with III tumors, indicating poorly defined tumor cells (PBC: *n* = 15 and CRC: *n* = 4).  Table 1 shows the characteristics of the study populations.

### 2.2. Enzyme Disaggregation of Tumor and Normal Tissues for Cell Isolation

Enzymatic digestion of fresh tumor tissues (TT) and paired, adjacent, nontumor normal tissues (NT) from breast and colorectal cancer patients was performed as described previously [4, 5]. Briefly, freshly resected tissues from cancer patients were mechanically cut into small pieces and then digested using an enzyme cocktail, consisting of 1 mg/ml collagenase (Sigma-Aldrich, Irvine, UK), 100 *μ*g/ml hyaluronidase type V (Sigma-Aldrich), and 30 IU/ml of deoxyribonuclease I (Sigma-Aldrich), and placed on a roll-over mixer at 37°C for 60 minutes. The cell suspension was then passed through a 100 *μ*m BD Falcon cell strainer (BD Biosciences, Oxford, UK) to remove debris and aggregates. Cells were then resuspended in RPMI-1640 enriched with 10% FCS and 1% Penicillin/Streptomycin after washing with RPMI-1640.

### 2.3. Staining of Whole Blood and Tissue-Infiltrating Immune Cells for Flow Cytometric Analyses

Fresh whole blood and isolated cells from freshly resected NT and TT were stained for phenotypical analyses as described previously [4, 5]. Briefly, cells were stained using myeloid markers (anti-human CD33, CD11b, HLA-DR, CD14, and CD15). Cells were then fixed and permeabilized using Fixation Permeabilization Buffer (eBioscience, San Diego, USA) to stain with anti-human arginase 1 (R&D Systems, Minneapolis, USA) antibody for intracellular staining. Samples were analyzed on BD FACSCanto II flow cytometer (BD Bioscience, San Jose, USA).

Immune cells extracted from ED were first blocked for Fc receptor using FcR Blocker (Miltenyi Biotec, Bergisch Gladbach, Germany), and 7AAD viability dye (eBioscience) was used to gate live cells. Cells were then stained with myeloid markers, and the flow cytometric data were analyzed on BD FACSuite software (BD Biosciences).

### 2.4. Population Calculation and Statistical Analyses

Each subpopulation of cells identified from flow cytometric analyses was compared within the three study groups by comparing the relative percentage of each subpopulation from the respective parent cell population and also by comparing the calculated percentages of each subpopulation. Statistical analyses were performed using GraphPad Prism 5.0 software (GraphPad Software, La Jolla, USA). One-way ANOVA/Kruskal-Wallis tests followed by paired/Wilcoxon matched-pairs signed-rank test or unpaired/Mann–Whitney tests were used to examine the differences within groups or between groups, respectively. *p* value of ≤0.05 was considered statistically significant. The data are presented as means ± SEM.

## 3. Results

### 3.1. CRC Patients Have Significantly Higher Levels of Circulating GMC Than PBC Patients and HD

We have previously reported levels of circulating myeloid cells in 23 PBC and 20 CRC patients and the levels of infiltrating myeloid cells in 7 PBC and 9 CRC patients [4, 5] using flow cytometric analyses. In this report, we have included data from some additional PBC patients (circulating myeloid cells; *n* = 30 and myeloid cells in TME; *n* = 9) and CRC patients (circulating myeloid cells; *n* = 20 and myeloid cells in TME; *n* = 11) and compared the levels between cancer patients and also with circulating myeloid cell levels in HD (*n* = 21).

**Figure 1:**
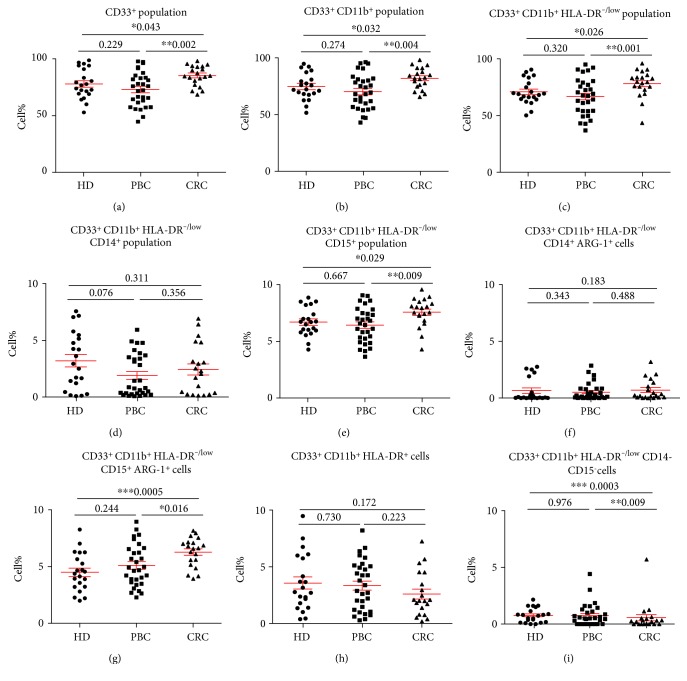
Comparison of different circulating myeloid cell subsets between HD and cancer patients. Peripheral blood from 21 HD, 30 PBC patients, and 20 CRC patients was stained for myeloid cell markers. Scatter plots show the mean percentages ± SEM of CD33^+^ cells gated on all cells (a), CD33^+^CD11b^+^ cells gated on CD33^+^ cells (b), CD33^+^ CD11b^+^HLA-DR^−/low^ cells (c), CD33^+^CD11b^+^HLA-DR^−/low^CD14^+^ MMC (d), CD33^+^CD11b^+^ HLA-DR^−/low^CD15^+^ GMC (e), ARG1 expression in MMC (f), ARG1 expression in GMC (g), CD33^+^ CD11b^+^HLA-DR^+^ APC (h), and CD33^+^ CD11b^+^HLA-DR^−/low^CD14^−^CD15^−^IMC (i) gated on the respective parent population in the three study groups. ∗ indicates *p* value of ≤0.05, ∗∗ indicates *p* value of ≤0.01, and ∗∗∗ indicates *p* value of ≤0.001.

There were significant differences in the levels of CD33^+^ myeloid cells between the three study groups (one-way ANOVA test; *p* = 0.007, Figure 1(a)). We found that CRC patients have significantly higher levels of CD33^+^ cells than both PBC and HD (CRC: 85.28 ± 1.98% versus PBC: 73.03 ± 2.73% and HD: 77.96 ± 2.85%, Figure 1(a)). This expansion of myeloid cells in CRC patients was confirmed further with the addition of another myeloid marker, CD11b, as CRC patients had significantly higher levels of circulating CD33^+^CD11b^+^ cells than HD and PBC (CRC: 81.92 ± 2.07 versus PBC: 70.20 ± 2.79 and HD: 74.59 ± 2.56, Figure 1(b)). There was no difference in the levels of CD33^+^ and CD33^+^CD11b^+^ cells between PBC and HD (Figures 1(a) and 1(b)). We then looked for HLA-DR expression within CD33^+^CD11b^+^ populations between the study cohorts. HLA-DR is a MHC class II molecule and is expressed on antigen-presenting cells (APC). We found that CRC patients have significantly higher levels of CD33^+^CD11b^+^HLA-DR^−/low^ cells than HD and PBC (CRC: 78.22 ± 2.65 versus PBC: 66.86 ± 2.96 and HD: 70.94 ± 2.40, Figure 1(c)).

We then analyzed CD15 and CD14 expression within the CD33^+^CD11b^+^HLA-DR^−/low^ populations to further investigate the morphology of the expanded circulating myeloid cells in the study cohorts. We did not find any significant differences in CD14^+^ cells in cancer patients compared to HD (Kruskal-Wallis test; *p* = 0.222, CRC: 2.4 ± 0.5; PBC: 2.3 ± 0.4; and HD: 3.4 ± 0.6; Figure 1(d)). However, there were significant differences in CD15 expression between the three study groups (Kruskal-Wallis test; *p* = 0.021). CRC patients showed significantly higher levels of GMC in peripheral blood compared to HD (CRC: 75.8 ± 2.8, HD: 64.1 ± 4.0, *p* = 0.029) and PBC (PBC: 66.8 ± 3.3, *p* = 0.009) as shown in Figure 1(e).

We used established markers for MDSC in these studies and investigated arginase 1 (ARG1) expression, indicative of their suppressive potential. There were no significant differences in the levels of ARG1-expressing MMC in the three study groups (HD: 0.6 ± 0.2, CRC: 0.7 ± 0.2, and PBC: 0.6 ± 0.2; Figure 1(f)). However, there were significant differences in the expression of ARG1 by GMC cells between the three study groups (one-way ANOVA test; *p* = 0.004). Levels of ARG1-expressing GMC cells were significantly higher in the peripheral blood of CRC patients than in HD (CRC: 63.1 ± 2.9 versus HD: 43.2 ± 4.1) and PBC patients (PBC: 52.2 ± 4.4; Figure 1(g)).

Myeloid cells which express CD33 and CD11b but lack HLA-DR, CD14, and CD15 expression were identified as IMC, as these cells are halted at different stages of differentiation and maturation. We compared the levels of these cells in the periphery between CRC and PBC and found that CRC patients have significantly lower levels of circulating IMC as compared to both HD and PBC (HD; 0.77 ± 0.48 versus PBC; 0.77 ± 0.18 versus CRC; 0.16 ± 0.06, Figure 1(i)).

### 3.2. Levels of GMC Are Higher in Colon Tissue Compared to Breast Tissue

**Figure 2:**
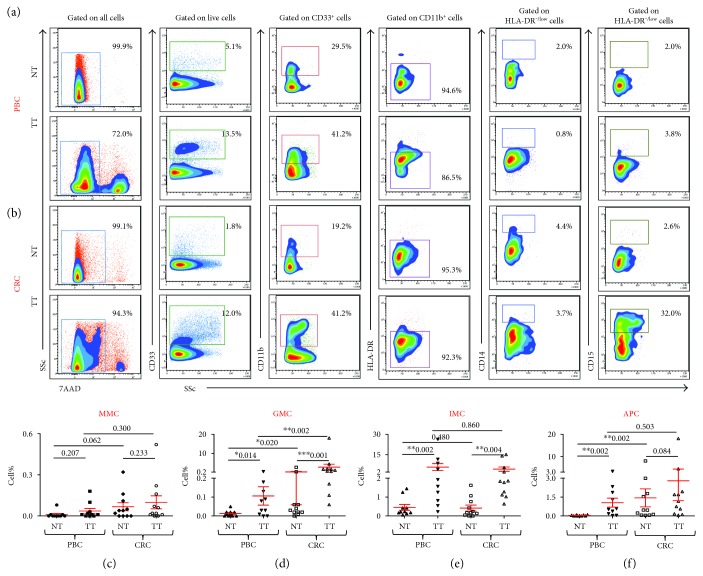
Levels of tumor-infiltrating myeloid cells in breast and colorectal cancer and paired, adjacent, nontumor tissue. Representative flow cytometric plots showing the levels of different subsets of myeloid cells in normal tissue (NT) and corresponding tumor tissues (TT) of 10 PBC (a) and 11 CRC patients (b). Scatter plots showing the mean percentages ± SEM of relative percentages of MMC (c), GMC (d), IMC (e), and APC (f) in NT and TT of PBC and CRC patients. ∗ indicates *p* value of ≤0.05, ∗∗ indicates *p* value of ≤0.01, and ∗∗∗ indicates *p* value of ≤0.001.

We compared levels of tumor-infiltrating myeloid cells in PBC and CRC patients. Representative flow cytometric plots for their levels in NT and TT of PBC and CRC patients are shown in Figures 2, a and 2, b. Although, there were no differences in the levels of MMC between PBC and CRC patients, cells isolated from normal colon tissue had higher levels of MMC than cells from normal breast tissue but the data did not reach statistical significance (*p* = 0.062, Figure 2, c). Interestingly, in addition to our previous findings that GMC and IMC are expanded in TME of PBC and CRC, we found that the levels of GMC in CRC NT are significantly higher than in PBC NT and although GMC are expanded in the TME of both cancers, levels of GMC in TME of CRC were significantly higher than in PBC (CRC NT; 0.30 ± 0.24, TT; 2.79 ± 1.58 versus PBC NT; 0.01±, TT; 0.11, Figure 2, d). IMC levels between the two study groups showed similar expansion compared to corresponding NT (Figure 2, e).

### 3.3. APC Are Expanded in Breast Cancer Tissue Compared to Normal Tissue, but the Levels of Infiltrating APC Are Lower Than Colon Tissue

We have previously reported that HLA-DR^+^ APC are expanded in breast cancer TME [5] but did not analyze the levels of APC in CRC TME before. Therefore, we analyzed and compared levels of APC of myeloid lineage between normal breast and colon tissue and the corresponding tumor tissues. Interestingly, we found that there is no significant expansion of the levels of tumor-infiltrating APC in CRC patients, but the levels of APC in normal colon tissue are significantly higher than in breast tumor tissue (CRC NT; 1.43 ± 0.72, TT; 2.79 ± 1.58 versus PBC NT; 0.04 ± 0.01, TT; 1.06 ± 0.36, Figure 2, f).

### 3.4. Circulating ARG1-Expressing GMC Are Higher in CRC Patients with Poorly Differentiated Tumors, but Myeloid Cell Levels Are Not Associated with Disease Stage or Tumor Histological Grade in PBC Patients

**Figure 3:**
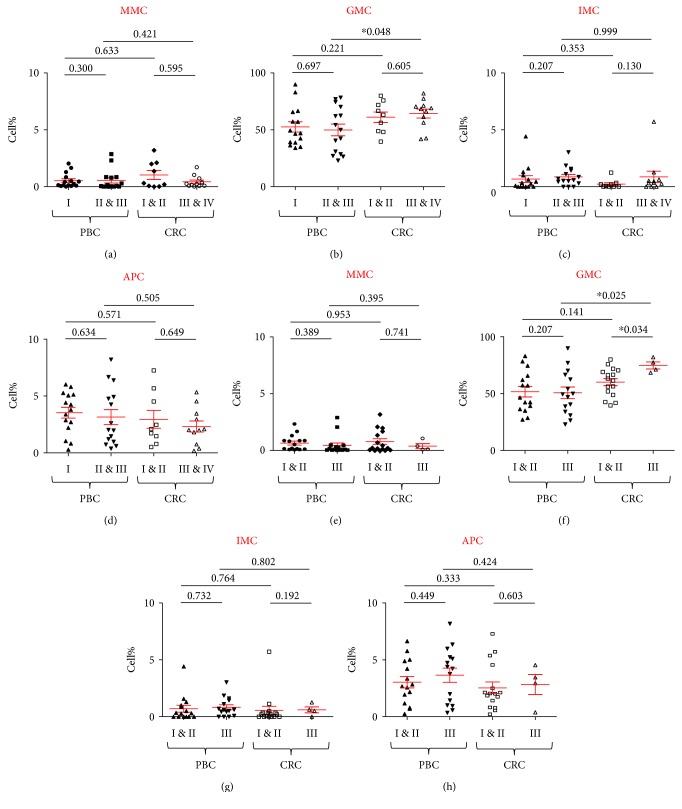
Comparison of circulating myeloid cells in cancer patients based on disease stage and the histological grading of tumors. Scatter plots showing mean ± SEM of levels of circulating myeloid cells in PBC with stage I (*n* = 15), compared with PBC with II and III (*n* = 15) and CRC with stage I and II (*n* = 9) and stage III and IV (*n* = 11) of MMC (a), GMC (b), IMC (c), and APC (d). Scatter plots showing mean ± SEM for levels of circulating myeloid cells in PBC patients with tumor histological grade I and II (*n* = 15) with grade III (*n* = 15) and CRC patients with grade I and II (*n* = 16) and grade III tumors (*n* = 4) of MMC (e), GMC (f), IMC (g), and APC (h). ∗ indicates *p* value of ≤0.05.

Next, we wanted to find out if the levels of myeloid cells in peripheral blood and tumor microenvironment of CRC and PBC could potentially correlate with tumor stage or tumor histological grade. Patient samples were divided based on TNM stage and tumor histological grade to compare the levels of myeloid cells between them. There was no significant correlation between the levels of tissue-infiltrating myeloid cells and tumor stage or grade for CRC and PBC patients, possibly due to the limited sample size (data not shown). For peripheral blood, CRC patients with tumor stage I and II (*n* = 9) were compared with tumor stage III and IV patients (*n* = 11), while PBC patients with stage I (*n* = 15) were compared with stage II and III cancer patients (*n* = 15). We found that CRC patients with advanced stage disease have significantly higher levels of circulating GMC than PBC with advanced disease (Figure 3(b)). There were no significant differences in the levels of other subsets of myeloid cell levels when we compared different tumor stages within peripheral blood samples from CRC and PBC (Figures 3(a), 3(c), and 3(d)).

We found significant differences in the levels of circulating myeloid cells in CRC patients when categorized based on the histological grade of differentiation of tumor cells (Figures 3(e), 3(f), 3(g), and 3(h)). Patients with histological grade I and II cancers had well-to-moderately defined tumor cells, while grade III patients presented with poorly differentiated tumor cells. CRC patients with high-grade III tumors (*n* = 4) had significantly higher levels of circulating GMC levels compared to patients with grade I and II tumors (CRC grade III: 74.8 ± 3.1 versus CRC grade I and II: 60.1 ± 3.1, Figure 3(f)). Moreover, the levels of circulating MMC, IMC, and APC did not correlate with tumor histological grade in both cancer cohorts. Noteworthy, these correlations were investigated in a small sample size and they should be further confirmed in larger studies.

## 4. Discussion

The TME contains a mixture of cells of lymphoid and myeloid lineages, comprising of both innate and adaptive immune cells. Tumor-infiltrating myeloid cellular populations include granulocytes (neutrophils, basophils, and eosinophils), Tie2-expressing monocytes, dendritic cells (DC), tumor-associated macrophages, IMC, and MDSC [8]. Expansion of infiltrating lymphocytes in cancers is often associated with antitumor response and improved clinical outcome [9]. While cancer treatment strategies like adoptive T cell therapies, which involve transferring in vitro expanded autologous cytotoxic T cells to eradicate tumor cells [10], aim to elevate the levels of infiltrating lymphocytes, MDSC are recognized as key checkpoint which favor evasion of immune response against tumor and are targeted to reduce their levels in the TME [11]. High neutrophil to lymphocyte ratio (NLR) has been recognized as a prognostic indicator, associated with patients with advanced stage disease in various malignancies [12]. Elevated levels of circulating MDSC inversely correlated with lymphocyte count and showed strong correlation with NLR in CRC patients [13].

We have previously reported an expansion of infiltrating myeloid cells in breast and colorectal cancers compared to normal tissue milieu [4, 5]. These cells exhibited granulocytic and immature myeloid cell phenotypes [14]. In the present study, we compared the levels of these cells between breast and colorectal cancers since these cancers have distinct pathogenesis as the former primarily results due to incarnation towards certain risk factors [15] and the latter primarily presents as an advanced inflammatory disease [16]. We looked for differences in the levels of circulating myeloid cells between cancer patients and HD first. MDSC are a heterogeneous population of cells and therefore there are no definite markers for MDSC recognition [3]. Various groups have identified MDSC based on a combination of myeloid markers and exclusion of cells of lymphoid lineages. We previously highlighted why we opted to use fresh whole blood for all our analyses on circulating MDSC levels, mainly to prevent loss of certain MDSC subsets when analyzing peripheral blood mononuclear cells (PBMC) in place of fresh whole blood [4]. In line with previous studies [13, 17–20], we also reported an expansion of circulating myeloid cells in CRC patients [4]. Additionally, we reported that myeloid cells are not expanded in periphery of breast cancer patients [5]. In the present analyses, we have established further that CRC patients have higher levels of myeloid cells in circulation than breast cancer patients also.

The close phenotypical and functional proximity of tumor-associated neutrophils (TAN) with G-MDSC [21] and identifying mature monocytes with M-MDSC has made it challenging to differentiate between them without exploiting the suppressive characteristics of these cells. MDSC can be, therefore, functionally categorized by their characteristic T cell suppression through arginase I (ARG1) expression and inducible nitric oxide synthase expression [3, 22]. We have previously reported high ARG1 expression in both circulating GMC and MMC [4, 5], and expansion of ARG1^+^ GMC in CRC compared to PBC in the present study shows the potential immunosuppressive role of myeloid cells in circulation in CRC patients.

APC have a pivotal role in antitumor immune response. Immunotherapeutic approach through the introduction of a tumor-associated antigen with an adjuvant to activate T cells *in vivo* is a novel approach [23]. Sipuleucel-T was the first therapeutic agent approved by the Food and Drug Administration (FDA) in 2010 designed to promote DC activation in prostate cancer patients [24]. Adoptive T cell therapies aim to expand and stimulate autologous T cell *in vivo* through APC to reintroduce in cancer patients to elicit potent immune response [25]. These modified T cells have high affinity for MHC complexes and have shown promising effects in cancer patients [25]. In our study, we have identified APC based on expression of MHC class II molecule, HLA-DR. We found that although HLA-DR^+^ APC are not expanded in the peripheral blood of CRC and PBC, they are higher in the TME of both cancers. Interestingly, we found that levels of APC in normal colon tissue are higher than in normal breast tissue. Intestinal APC which include DC and macrophage are an integral part of the innate mucosal system maintaining tolerance against food antigens and intestinal microbiota [26]. Moreover, DC from breast cancer biopsies were found to be distinct from macrophages and presented with remarkable cytotoxic T cell activation capabilities [27].

Tumor histological grade and disease stage based on TNM staging criteria are well-established tools to predict the clinical outcome in various cancers [28]. In a study of 1239 CRC patients, it was reported that tumor histology grade for differentiation correlated significantly with the TNM stage [29]. Similarly, numerous studies have laid emphasis on the significance of Nottingham histological grade in accurately predicting tumor behavior in breast cancer patients [30]. In our previous studies, we reported that expansion of peripheral GMC in CRC patients correlated with advanced stage and histological grade, which suggested their role in tumor progression [4]; however, no such correlation was observed in the levels of circulating myeloid cells in PBC [5]. We found that CRC patients with poorly differentiated tumors have significantly higher levels of GMC than those with well-defined tumors and also breast cancer patients, regardless of their tumor histological presentation. Therefore, our data suggest that myeloid cell levels in PBC are independent of patient's clinical presentation as reflected by the tumor grade and disease stage.

## 5. Conclusion

In conclusion, further to our previous findings of elevated levels of GMC and IMC in circulation and in the TME of CRC patients [4] and an expansion of IMC and GMC in the TME of PBC patients which was not reflected in circulation [5], in this study, we report that the GMC expansion in CRC patients is significantly higher than in PBC patients. Our results therefore suggest that CRC patients may benefit more from the development of therapeutic agents to promote myeloid cell differentiation or inhibition, which ultimately helps in reversal of immune suppression in most human cancers [31]. Additionally, our findings of higher levels of APC in the TME of CRC and PBC patients can be useful in the development of novel active immunotherapeutic approaches, tailored to improve tumor-associated antigen presentation to cytotoxic T cells.

## Figures and Tables

**Table 1:** Characteristic features of study subpopulations.

	HD	PBC	CRC
Number	21	30	20
Gender (male : female)	9 : 12	0 : 30	13 : 7
TNM stage			
I		15 (6)^∗^	2 (1)^∗^
II		11 (1)^∗^	7 (4)^∗^
III		4 (3)^∗^	10 (5)^∗^
IV		0 (0)^∗^	1 (1)^∗^
Histological grade			
Well/moderate		15 (5)^∗^	16 (10)^∗^
Poor/undifferentiated		15 (5)^∗^	4 (1)^∗^

HD: healthy donors; PBC: primary breast cancer patients; CRC: colorectal cancer patients. ^∗^Samples taken from patients for investigating tissue-infiltrating immune cells.
